# Supplementary material for: MetaRibo-Seq measures translation in microbiomes
Source: Nat Commun. 2020 Jun 29;11:3268. doi: 10.1038/s41467-020-17081-z (PMC7324362; doi:10.1038/s41467-020-17081-z)
Supplement: Supplementary file 10 — Supplementary Data 7 [file 41467_2020_17081_MOESM10_ESM.zip › File2/Confidence_VeryHigh_Taxonomy/116254_out.krona.html]

Javascript must be enabled to view this page.

members
magnitude
magnitudeUnassigned
count
unassigned
taxon
rank

116254\_out

6

10239
1
superkingdom

1
28883
order

10699
1
family

SRS020869\_contig\_number\_contig-100\_2828.38554

superkingdom
2
3

phylum
1239
3

3
186801
class

order
186802
3

family
2
31979

1485
2
genus


SRS019068\_contig\_number\_92426
species
2293035
1


SRS015431\_contig\_number\_6309
1
2293012
species

186806
1
family

1
1730
genus


SRS042284\_contig\_number\_6612
species
39485
1

2

SRS045645\_contig\_number\_3513SRS048164\_contig\_number\_contig-100\_8224.8224
